# *Candida albicans*, a distinctive fungal model for cellular aging study

**DOI:** 10.1111/j.1474-9726.2008.00424.x

**Published:** 2008-10

**Authors:** Xiao-Hong Fu, Fei-Long Meng, Yan Hu, Jin-Qiu Zhou

**Affiliations:** Max-Planck Junior Research Group in the State Key Laboratory of Molecular Biology, Institute of Biochemistry and Cell Biology, Shanghai Institutes for Biological Sciences, Chinese Academy of Sciences; Graduate School of Chinese Academy of SciencesShanghai 200031, China

**Keywords:** aging, *Candida albicans*, extra-chromosomal rDNA molecules, model, old cell preparation, *SIR2*

## Abstract

The unicellular eukaryotic organisms represent the popular model systems to understand aging in eukaryotes. *Candida albicans*, a polymorphic fungus, appears to be another distinctive unicellular aging model in addition to the budding yeast *Saccharomyces cerevisiae* and fission yeast *Schizosaccharomyces pombe*. The two types of *Candida* cells, yeast (blastospore) form and hyphal (filamentous) form, have similar replicative lifespan. Taking the advantage of morphologic changes, we are able to obtain cells of different ages. Old *Candida* cells tend to accumulate glycogen and oxidatively damaged proteins. Deletion of the *SIR2* gene causes a decrease of lifespan, while insertion of an extra copy of *SIR2* extends lifespan, indicating that like in *S. cerevisiae*, Sir2 regulates cellular aging in *C. albicans*. Interestingly, Sir2 deletion does not result in the accumulation of extra-chromosomal rDNA molecules, but influences the retention of oxidized proteins in mother cells, suggesting that the extra-chromosomal rDNA molecules may not be associated with cellular aging in *C. albicans*. This novel aging model, which allows efficient large-scale isolation of old cells, may facilitate biochemical characterizations and genomics/proteomics studies of cellular aging, and help to verify the aging pathways observed in other organisms including *S. cerevisiae*.

## Introduction

Aging is usually defined as the progressive loss of function accompanied by decreasing fertility and increasing mortality with advancing age (Kirkwood & Austad, 2000). Lifespan regulation is evolutionarily conserved, and found in various species ranging from eukaryotic multicellular (e.g. humans) to unicellular (e.g. yeast), and to prokaryotic (e.g. *Escherichia coli*) organisms (Stewart *et al*., 2005). In unicellular species, cellular aging and organismal aging coincide. Previous studies in several model organisms have described many aging-related phenotypes, including morphological changes, accumulation of oxidatively damaged DNA and proteins, shortened telomeres, enriched extra-chromosomal rDNA circles (ERCs), etc. (reviewed in Sinclair *et al*., 1998; Balaban *et al*., 2005; Kirkwood, 2005; Lombard *et al*., 2005). Nowadays, the elucidation of the molecular mechanism(s) in aging is of great interest to biologist.

The budding yeast *Saccharomyces cerevisiae*, which divides asymmetrically, is a unicellular eukaryotic organism with a short and easily studied lifespan, and enables researchers to chase an individual cell through many cell divisions. The yeast cells undergo a limited number of cell divisions before senescence, and small budding daughter cells come from the larger aging mother cells in most of the lifespan. The total number of daughter cells produced by a mother cell prior to senescence is defined as the replicative lifespan of the mother cell (Mortimer & Johnston, 1959). Aging in yeasts *S. cerevisiae* and *Schizosaccharomyces pombe* has also been measured as the length of time a population stay alive; it is referred to as chronological aging (Fabrizio & Longo, 2003; Roux *et al*., 2006).

Recent studies reveal that some of the molecular mechanisms of aging existing in higher eukaryotes have also been unraveled in *S. cerevisiae*. For example, the human WRN helicase defect causes the premature aging Werner syndrome (Epstein *et al*., 1966), while mutation of Sgs1 helicase, the WRN homolog in yeast, causes the shortening of replicative lifespan (Sinclair *et al*., 1997). Calorie restriction, which significantly promotes longevity in various organisms including worms, flies, fish and mammals, also lengthens yeast lifespan (Weindruch & Walford, 1988; Jiang *et al*., 2000; Lin *et al*., 2000; Lane *et al*., 2001). Deletion of Sir2 histone deacetylase decreases the replicative longevity of yeast, whereas an extra copy of *SIR2* integrated into the genome results in longer replicative lifespan (Kaeberlein *et al*., 1999). Similarly, increasing the dosage of Sir2.1 in *Caenorhabditis elegans* (the homologue of yeast Sir2) or *Drosaphila* Sir2 extends lifespan (Tissenbaum & Guarente, 2001; Rogina & Helfand, 2004). So far, the mechanism by which Sir2 regulates lifespan is not well understood. *Saccharomyces* Sir2 has been shown to act at blocked replication forks in the rDNA to prevent DNA breaks, homologous recombination and ERC formation in a Fob1-mediated pathway (Kaeberlein *et al*., 1999). Therefore, it is suggested that ERCs are a cause of yeast aging (Sinclair & Guarente, 1997), and Sir2 plays a specific role in promoting longevity.

Since the budding yeast *S. cerevisiae* has been developed as an advantageous model of aging research, the development of techniques for large-scale isolation of old cells is extremely required in any biochemical or high-throughput analysis. As this problem is of major interest, several methods have been established in *S. cerevisiae* for obtaining large quantities of old cells (Park *et al*., 2002), including yeast ‘baby machine’ (Helmstetter, 1991), centrifugation elutriation (Woldringh *et al*., 1995), sucrose gradient centrifugation (Egilmez *et al*., 1990), fluorescence-activated cell sorting (Sloat & Pringle, 1978) and magnetic sorting (Smeal *et al*., 1996). However, these approaches seem costly and laborious because (i) the mother and daughter cells are similar in shape; (ii) the proportion of old cells in any growing culture of logarithmic phase is miniscule (Park *et al*., 2002); (iii) as *S. cerevisiae* yeast mother cells grow old, they enlarge and tend to produce large and short-lived daughter cells (Kennedy *et al*., 1994), and at last several divisions, daughter and mother cells are usually indistinguishable in size and can hardly separate from each other (Kennedy *et al*., 1995). Hence, we are encouraged to develop another model, *Candida albicans*, for aging study.

*Candida albicans*, which diverges from *S. cerevisiae* over 140 million years ago (Berman & Sudbery, 2002), has been evolved to be a polymorphic fungus and exists as a commensal of warm-blooded animals including humans. It can grow in different ways: proliferating in yeast form and forming filamentous hyphal cells that can give birth to new mycelia or yeast form (ellipsoidal or blastospore) daughters (Molero *et al*., 1998). The growth form can be controlled by changing culture conditions including temperature, pH and nutrient composition (Soll, 1986). *C. albicans* usually exists as a diploid and parasexual organism, which is different from *S. cerevisiae*. As these interesting features are concerned, we anticipated to establish *C. albicans* as a new aging model complementary to *S. cerevisiae* and *S. pombe*. The complete genome sequencing has revealed that many *C. albicans* open reading frames have obvious *S. cerevisiae* homologs and the well-established tools for molecular-genetic manipulations make it feasible to set up *C. albicans* as a model organism.

In the current study, we observed that *C. albicans* cells, like *S. cerevisiae* ones, have a finite lifespan. We have established a method to prepare a large number of old cells, facilitating the high-throughput analysis of cellular aging in *C. albicans*. We also characterized the old cells and found out that glycogen and oxidatively damaged proteins accumulate during the replicative aging of *C. albicans*. In addition, Sir2 regulates *Candida* lifespan in a dose-dependent manner, but the extra-chromosomal rDNA molecules show no obvious correlations with Sir2 dosage, suggesting that unlike in *S. cerevisiae*, extra-chromosomal rDNA molecules may not be the marker of aging in *C. albicans*.

## Results

### Both yeast and hyphal form of *C. albicans* cells have finite lifespan

**Fig. 1:**
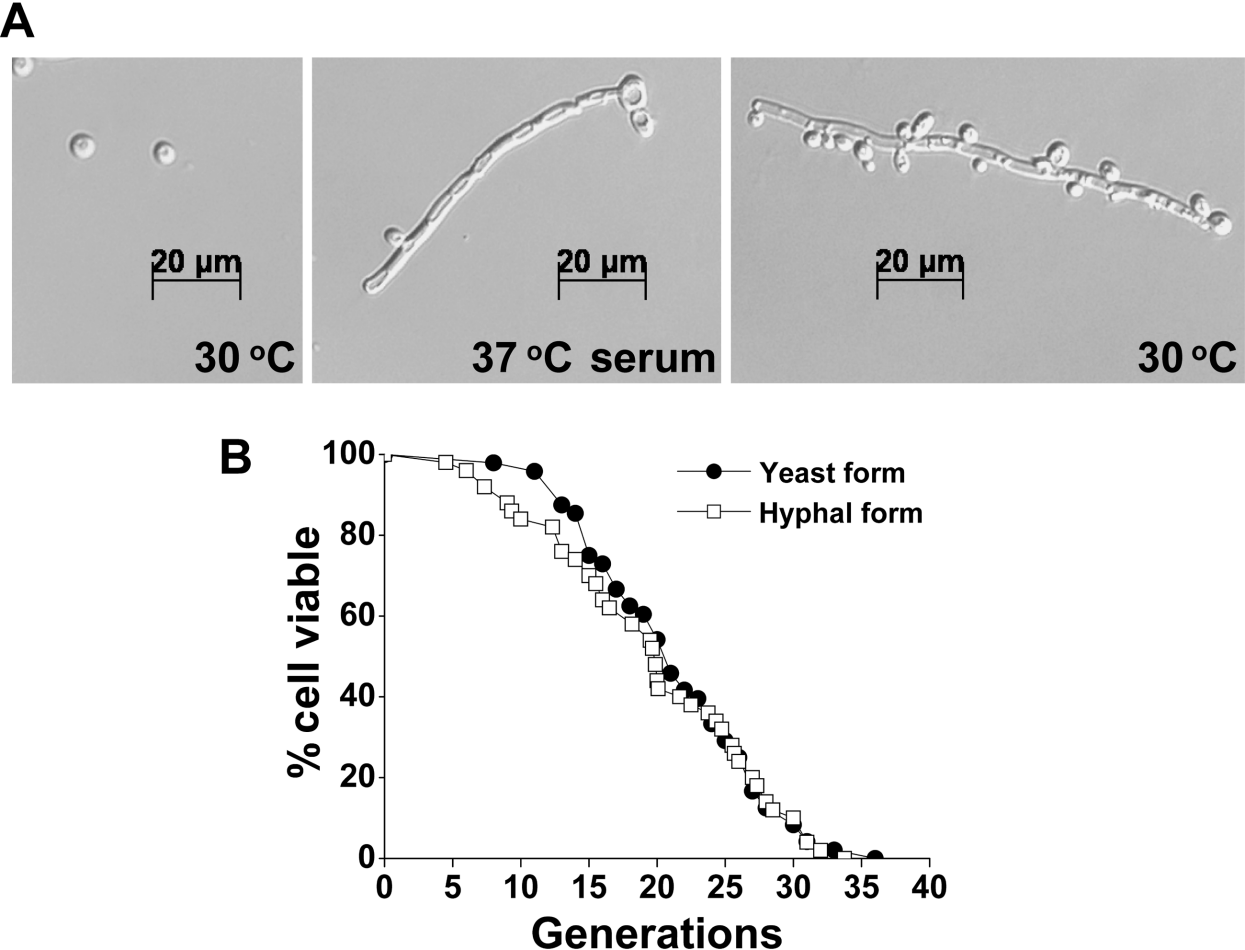
Both yeast and hyphal form cells share similar replicative lifespan. (A) Different forms of *Candida albicans*. Left panel, *C. albicans* cells of yeast form cultured at 30 °C in YPD; middle panel, *C. albicans* cells of hyphal form induced by serum at 37 °C; right panel, yeast daughter cells produced by hyphal mother cells at 30 °C in the absence of serum. Photos were taken with a Zeiss Axioplan 2 microscope. (B) Lifespan analysis of yeast and hyphal cells. Mean lifespan and sample size are: yeast form 21.3 (*n* = 48), and hyphal form 19.3 (*n* = 54).

*Candida albicans* is a prevalent opportunistic fungal pathogen in humans, and can grow in budding form or filamentous form (Fig. 1A). Like *S. cerevisiae*, *C. albicans* cells of yeast form propagate by budding at 30 °C (Fig. 1A, left panel). In response to serum at 37–40 °C, the yeast form cells can be induced to filamentous hyphae that usually contain a few cells in tandem (Fig. 1A, middle panel). The filamentous hyphal cells can also give rise to yeast form daughters when they are grown at 30 °C in the absence of serum (Fig. 1A, right panel) (Molero *et al*., 1998).

**Fig. 2:**
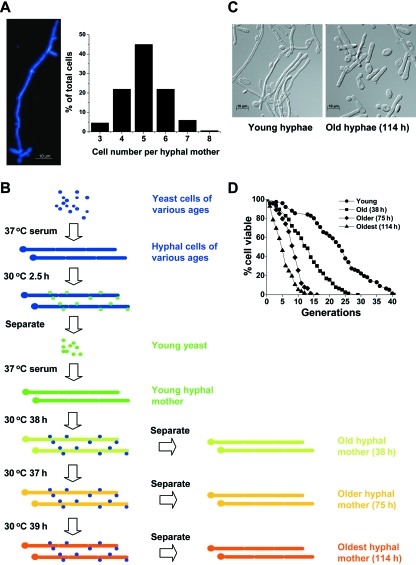
Large-scale separation of old and young cells. (A) Most of the hyphae induced under the experimental conditions contain four to six single hyphal cells. Left panel, cells stained by Calcofluor and photographed under microscope; right panel, statistical results. (B) Schematic procedures for large-scale preparation of old cells. (C) Morphological differences between old and young cells. Photos were taken with a Zeiss Axioplan 2 microscope using 10 × 100 magnification. (D) Lifespan analysis of hyphal cells separated at the time points of 0 h, 38 h, 75 h and 114 h, respectively. Mean lifespan and sample size are: 22.9 (*n* = 76), 13.1 (*n* = 76), 8.4 (*n* = 74) and 5.4 (*n* = 56), respectively.

To find out whether *C. albicans* cells have finite lifespan, we performed replicative lifespan assay of both yeast and hyphal form cells. Since yeast cells produce daughters by budding, we analyzed their replicative lifespan as described in *S. cerevisiae* (Kaeberlein *et al*., 2004). Wild-type cells of SC5314 strain have a mean lifespan of 19–23 generations (Fig. 1B and Fig. S4). To measure the lifespan of hyphal cells, we induced the growth of hyphal mothers containing four to six single cells in tandem that can produce yeast daughters (Fig. 2A). The total daughters produced by a hyphal mother were counted. The ratio of the total daughter number to the cell number of a hyphal mother represents the replicative lifespan of a single hypha cell. Figure 1B shows that hyphal form cells share similar replicative lifespan with yeast form cells, indicating the morphological change does not affect the lifespan. These results also demonstrate that both yeast and hyphal form cells have finite replicative lifespan. Our data suggest that *C. albicans* could be used as a model to study cellular aging.

### Dramatic morphological difference facilitates large-scale preparation of replicatively old cells

Biochemical investigation of the age-associated changes often requires a large-scale preparation of sufficiently pure and terminally senescent cells. However, old cells only account for a negligible fraction of a logarithmic growing culture. The isolation of replicatively aged cells has proved to be one of the most difficult tasks in yeast aging research. Since the hyphal cells of *C. albicans* can produce yeast form daughters, we thought that the dramatic difference in size between hyphal and yeast cells provided an opportunity to feasibly separate the old cells from their progenies. With this in mind, we established a method that allows separation of hyphal and yeast form cells based on sucrose gradient centrifugation. The separation efficiency was determined by the percentage of hyphal cells in the population. Most of the hyphae in our culturing system have four to six hyphal cells and one original yeast cell (Fig. 2A). Hyphal cells account for ∼80% of the population after separation, so we think the separation is efficient enough. The separated hyphal cells are shown in Fig. S1.

To separate the old hyphal mothers from their yeast daughters and study the aging associated phenotypes in *C. albicans*, we designed a procedure to obtain certain amount of old mother cells, schematically shown in Fig. 2B. Briefly, yeast form cells were incubated at 30 °C overnight, and hyphal cells were induced at 37 °C in the presence of serum for about 5 h. The hyphal cells in this culture should be at various replicative ages. The purified hyphae were then shifted back to YPD medium (1% yeast extract, 2% peptone, 2% glucose) to produce yeast cells at 30 °C for about 2.5 h. The relatively short time of culture ensured that the newborn yeast cells experienced no more than two cell divisions and could be regarded as young cells. These ‘synchronized’ young yeast cells were isolated from the culture by sucrose gradient centrifugation and then used to generate young hyphal mothers. When the hyphal mothers were cultured at 30 °C, they produced yeast form daughters and became older and older with prolonged culture time. Therefore, the hyphal cells are old cells and separated from yeast cells by sucrose gradient centrifugation (Fig. 2B). In this experiment, there are rare opportunities of young yeast daughter contamination in old hyphal mothers due to the dramatic size difference between hyphal and yeast form cells.

**Fig. 3:**
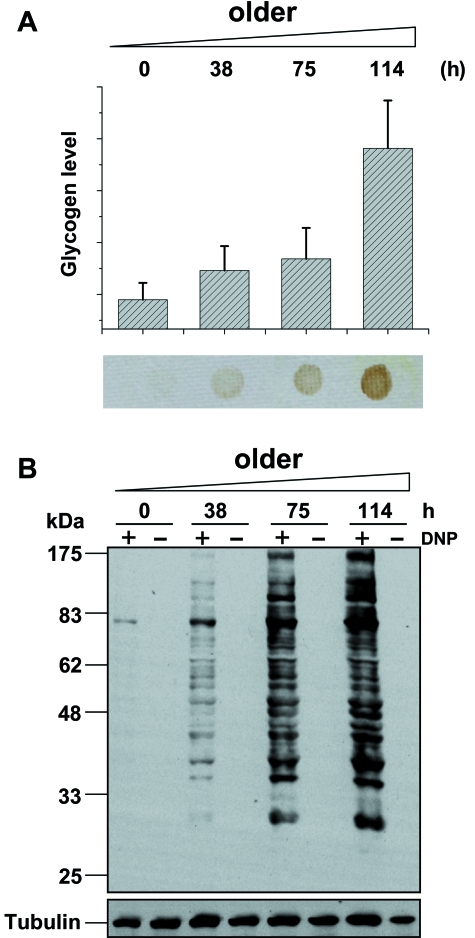
Accumulation of glycogen and oxidatively damaged proteins during *Candida* aging. (A) Examination and quantification of glycogen level by iodine staining. Error bars represent standard deviations of triple determinations. (B) Analysis of protein oxidation by anti-DNP immunoblotting. Total proteins were quantified by Bradford before loading. Tubulin served as an internal control.

In order to verify the separation strategy, we measured the replicative lifespan of the hyphal mothers separated at various time points (0 h, 38 h, 75 h and 114 h). As shown in Fig. 2D, the lifespan of the hyphal cells decreases along with growing old and the division capacity of the oldest cells is diminished largely, indicating that the hyphal mothers age gradually after they continuously give birth to yeast daughters, and the oldest cells we obtained are presumably not far from replicative senescence. In the following sections (Figs 3 and 5), we used the cell samples prepared at the same four time points. The old cells can also be distinguished from the young cells morphologically: hyphae are enlarged and broken into single cells (Fig. 2C). The procedures described here offer an opportunity to prepare old cells of different replicative ages in large-scale and examine the progressive changes associated with cellular aging.

### Glycogen and oxidatively damaged proteins accumulate during replicative aging of *C. albicans*

Glycogen accumulation has been described as a marker of aging, and reported in *S. cerevisiae* (Lin *et al*., 2001). Genomics study has also suggested that genes involved in gluconeogenesis and glycogen production are induced in old cells (Lesur & Campbell, 2004). We analyzed the glycogen level in *C. albicans* hyphal cells of different replicative ages, and the results showed that glycogen level increased during the aging period (Fig. 3A). The accumulation of glycogen in old cells may reflect a turnover of carbohydrates catabolism and energy metabolism.

It is increasingly evident that the oxidative modification of cellular components, including oxidation of proteins, lipids and nucleic acids, is another apparent phenotype in old cells over many species (Stadtman, 2006). To characterize the old *C. albicans* cells, we examined the overall oxidation of proteins according to the methods reported by Levine *et al*. (Levine *et al*., 1994; Cabiscol *et al*., 2000). The carbonyl groups (aldehydes and ketones) are introduced into oxidatively modified proteins irreversibly (Stadtman, 1992). Based on the reaction of carbonyl groups with 2,4-dinitrophenylhydrazine to form a 2,4-dinitrophenylhydrazone, we could immunodetect protein carbonylation using antibodies to the 2,4-dinitrophenyl moiety (Levine *et al*., 1994). Figure 3B shows that the level of carbonylated proteins was increasing remarkably as cells age, and the level of tubulin served as an internal control. These data demonstrate that oxidatively damaged proteins accumulate during replicative aging of *C. albicans*. Our results are consistent with the work in *S. cerevisiae* reported previously (Aguilaniu *et al*., 2003; Reverter-Branchat *et al*., 2004; Grzelak *et al*., 2006). It has been postulated that the age-related accumulation of oxidized proteins is a complex process that may integrate increases of reactive oxygen species generation rates, decreases in antioxidant activities or losses in the capacity to degrade oxidized proteins (Stadtman, 2006).

In addition to glycogen and oxidatively damaged proteins that accumulate in old *C. albicans* cells, we also examined telomere length and chromosome integrity by Southern blot and pulsed field gel electrophoresis (PFGE), respectively. No obvious differences between young and old cells have been observed (Figs S2 and S3).

### Lower glucose concentration delays cellular aging in *C. albicans*

**Fig. 4:**
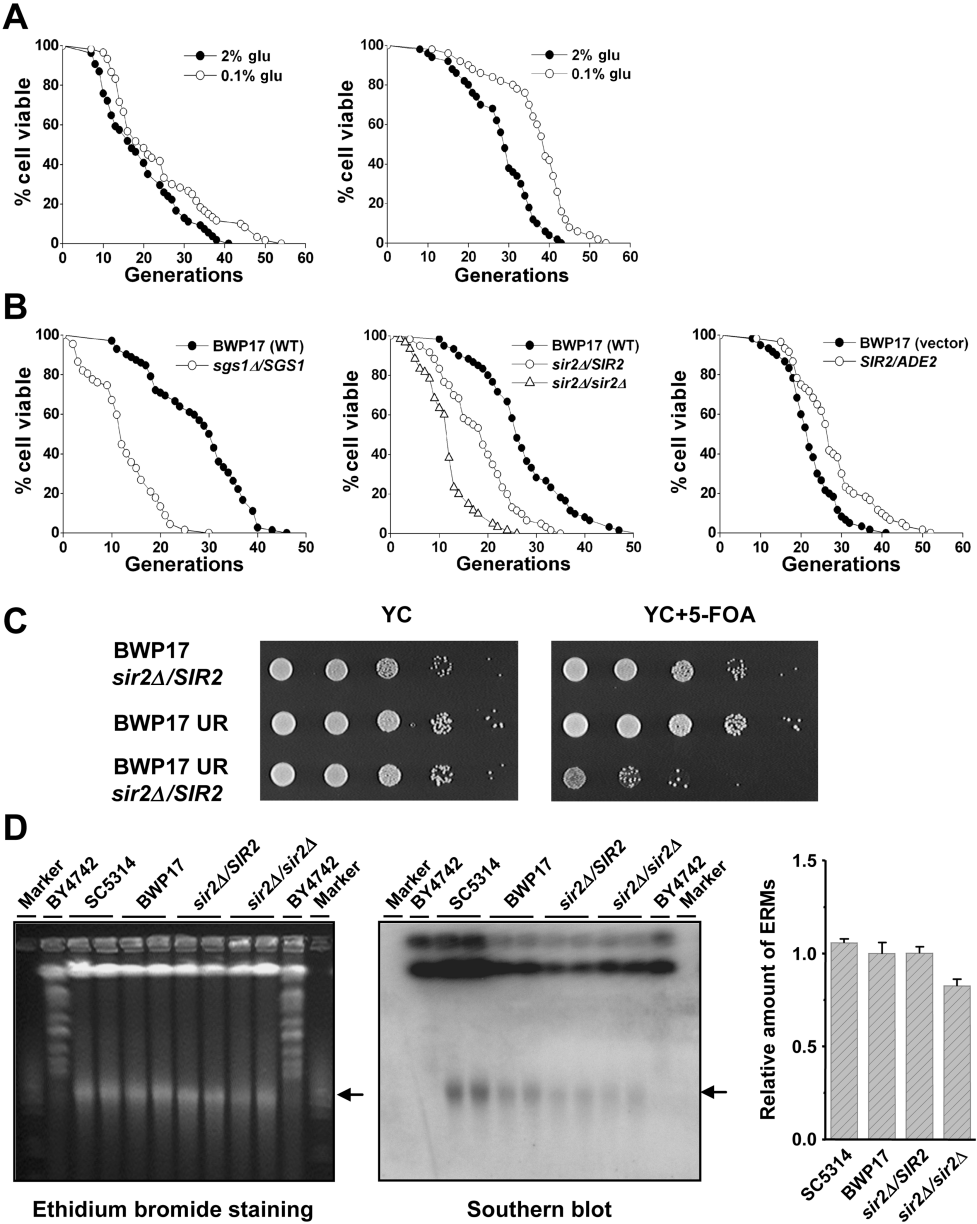
Characterization of *SIR2* function in *Candida* aging. (A) Lower glucose concentration lengthens the lifespan of *C. albicans*. Left panel, strain SC5314; right panel, strain BWP17. Mean lifespan and sample size of SC5314: 2% glucose 19.1 (*n* = 54), 0.1% glucose 23.7 (*n* = 60); BWP17: 2% glucose 28.0 (*n* = 50), 0.1% glucose 36.8 (*n* = 50). (B) Left panel, deletion of one copy of *SGS1* causes a shortened lifespan. Mean lifespan and sample size are: BWP17 (wild-type) 28.1 (*n* = 72), *sgs1Δ/SGS1* 12.6 (*n* = 67). Middle panel, reduced dosage of Sir2 decreases lifespan. Mean lifespan and sample size are: BWP17 (wild-type) 27.4 (*n* = 60), *sir2Δ/SIR2* 18.2 (*n* = 60), *sir2Δ/sir2Δ* 11.9 (*n* = 60). Right panel, insertion of an extra copy of *SIR2* results in lifespan extension. Mean lifespan and sample size are: BWP17 (vector) 22.4 (*n* = 60), *SIR2/ADE2* 27.8 (*n* = 60). (C) Haplo-insufficiency of *SIR2* causes a reduction of rDNA silence. Cells from logarithmic culture were 10-fold serially diluted, spotted onto plates, incubated at 30 °C for 2 days and photographed. The isogenic strains were labeled on the left. Left panel, cells grown on YC plate; right panel, cells grown on YC+5-FOA plate. BWP17 UR represents the strain with a *URA3* gene inserted in rDNA loci. (D) Detection of extra-chromosomal rDNA molecules using pulsed field gel electrophoresis. Left panel, ethidium bromide staining of the PFGE gel; middle panel, Southern blot examination with an rDNA probe. The isogenic strains were labeled on the top and each lane represents an independent clone of the corresponding strain. Marker, Low Range PFG marker (New England BioLabs); *Saccharomyces cerevisiae* (strain BY4742) chromosomes are used as another marker. Arrows indicate the extra-chromosomal rDNA molecules. Right panel shows the quantification of extra-chromosomal rDNA molecules (ERMs). The relative amount of ERMs in BWP17 strain was assigned a value of 1. Error bars represent standard deviations of two independent clones.

Calorie restriction extends lifespan in a variety of species and has been proved a general mechanism to enhance longevity (Weindruch & Walford, 1988; Masoro, 2002). In the yeast form cells of *C. albicans*, we examined the effect of low glucose concentration (Lin *et al*., 2000; Kaeberlein *et al*., 2002) on replicative lifespan. Cells grown in the presence of 0.1% glucose exhibited a longer lifespan in both SC5314 and BWP17 strains (Fig. 4A). Although the increase of lifespan in strain SC5314 seems weaker than that in strain BWP17 or *S. cerevisiae*, the calorie-restriction effect in strain SC5314 is significant because the *p*-value is less than 0.05. These data indicate that like in other species, calorie restriction extends lifespan in *C. albicans*. Notably, the two wild-type strains, SC5314 and BWP17, exhibited different lifespan: SC5314 is short-lived, while BWP17 is long-lived (Fig. S4). The divergence may be resulted from their different genetic background.

### Replicative lifespan of *C. albicans* correlates with the dosage of Sir2

It is known that the human Werner syndrome is caused by a defect in WRN helicase. Mutation of Sgs1 helicase, the WRN homolog, which is involved in DNA metabolism, causes the shortening of yeast lifespan (Sinclair *et al*., 1997). In order to study a *Candida* counterpart of the *SGS1* gene, we deleted one copy of *SGS1* in *C. albicans*, and measured the replicative lifespan of the mutant cells. The shortened lifespan of the *sgs1Δ/SGS1* heterozygote reveals a haplo-insufficiency, indicating that as in *S. cerevisiae*, Sgs1 is required for the regular lifespan of *C. albicans* (Fig. 4B, left panel).

Previous studies in *S. cerevisiae* demonstrate that Fob1/Sir2 pathway is involved in regulating cellular aging via affecting ERCs (Sinclair & Guarente, 1997; Kaeberlein *et al*., 1999). *sir2Δ* mutant exhibits shorter lifespan, whereas an extra copy of *SIR2* integrated into the genome results in longer lifespan (Kaeberlein *et al*., 1999). The deletion of *FOB1*, a gene required for blocking rDNA replication, suppresses rDNA recombination and ERC formation, extends lifespan in wild-type cells, and restores lifespan in *sir2* mutant cells (Defossez *et al*., 1999). Despite the fact that the Fob1 homolog in *C. albicans* has not been identified, the Sir2 homologue in *C. albicans* has been reported to be essential for the maintenance of chromosome stability (Perez-Martin *et al*., 1999). We suspected that Sir2 also affects *Candida* longevity. Because *C. albicans* always lives as a diploid, we examined the lifespan of *sir2Δ/SIR2* and *sir2Δ/sir2Δ* cells. Like *S. cerevisiae*, the reduced dosage of Sir2 resulted in a decrease of replicative lifespan (Fig. 4B, middle panel) (Kaeberlein *et al*., 1999). When an extra copy of *SIR2* was integrated into the genome at the *ADE2* locus, the lifespan increased compared with the insertion of the empty vector (Fig. 4B, right panel), suggesting that a conserved Sir2 pathway that regulates cellular aging exists in *C. albicans*. It is not clear why the introduction of a control empty vector at *ADE2* locus in BWP17 shortened its lifespan (Fig. S4A). One possible explanation is that the disruption of *ADE2* has a negative role on replicative lifespan.

### Extra-chromosomal rDNA molecules do not increase in *sir2Δ* mutants of *C. albicans*

Sir2 in *S. cerevisiae* has been indicated to affect the recombination of rDNA to regulate cell longevity (Guarente, 2000). Homologous recombination between adjacent rDNA repeats which locate on chromosome XII results in the formation of ERCs. Sir2 is essential for the silenced chromatin formation in rDNA loci (Bryk *et al*., 1997). In *C. albicans*, the rDNA units are clustered as tandem repeats on both chromosome R, and each unit is about 11.5 to 12.5 kb (Wickes *et al*., 1991; Iwaguchi *et al*., 1992; Rustchenko *et al*., 1993). The insertion of an exogenous *URA3* gene in rDNA loci did not affect the growth of cells in the 5’-FOA containing medium, indicating that the *URA3* gene was silenced as observed in *S. cerevisiae* (Fig. 4C, row 2). In contrast, single copy deletion of *SIR2* liberated the expression of the *URA3* gene, demonstrating a reduction of rDNA silencing (Fig. 4C, row 3).

The silenced chromatin inhibits homologous recombination between rDNA repeats, and reduces the formation of ERCs. Several lines of evidence suggest that the ERC accumulation may be a cause of cell aging in *S. cerevisiae*, and introduction of artificial ERCs shortens lifespan of yeast cells (Sinclair & Guarente, 1997). In *C. albicans*, the extra-chromosomal rDNA containing molecules have also been reported, but their size is ranging from 50 to 100 kb (Perez-Martin *et al*., 1999), which are much larger than the ones detected in *S. cerevisiae*. To investigate whether the extra-chromosomal rDNA molecules increases when the *SIR2* gene is deleted in *C. albicans*, we employed PFGE to separate the whole genome, and did Southern blot using an rDNA probe. As shown in Fig. 4D, the extra-chromosomal rDNA molecules were not increased in *sir2Δ/SIR2*or *sir2Δ/sir2Δ* mutant as compared with the wild-type strain BWP17. These data indicate that the decrease of lifespan in 2 mutant cells may not be associated with the amount of extra-chromosomal rDNA molecules.

Previous work by Perez-Martin *et al*. (1999) reported that extra-chromosomal rDNA molecules were not detected in wild-type strain 3153 A, but enriched in *sir2Δ/sir2Δ* cells of *C. albicans*. In our experiments, the extra-chromosomal rDNA molecules have been detected in all examined strains including the wild-type SC5314 cells (Fig. 4D). The discrepancy may be resulted from a strain-specific genetic background.

### Extra-chromosomal rDNA molecules do not accumulate in aging *Candida* cells

**Fig. 5:**
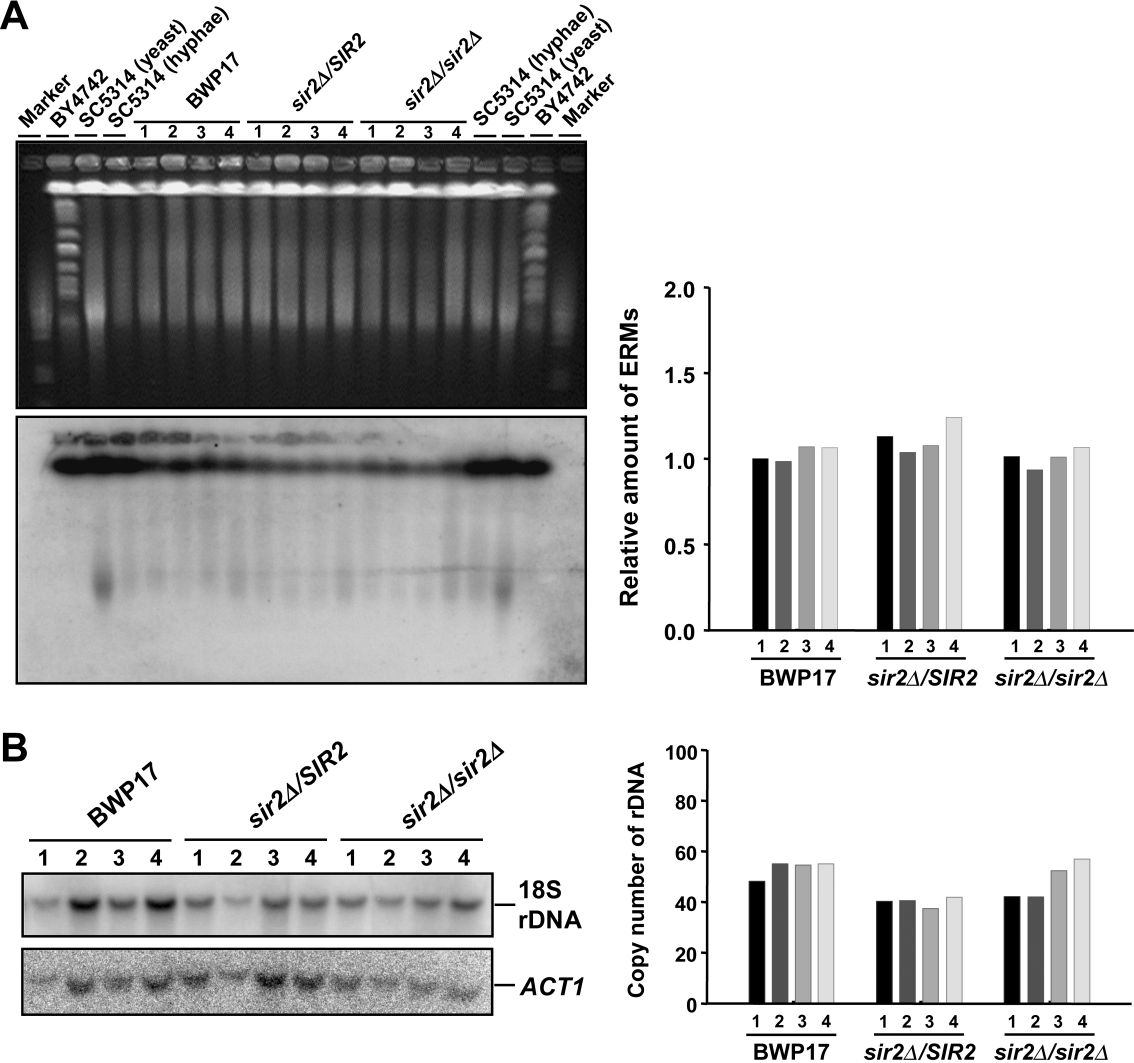
The extra-chromosomal rDNA molecules are not increased during *Candida* aging. (A) Detection of extra-chromosomal rDNA molecules during replicative aging. Upper left, ethidium bromide staining of the gel; lower left, Southern blot examination with an rDNA probe. Marker, Low Range PFG marker (New England BioLabs); *Saccharomyces cerevisiae* BY4742 chromosomes served as another marker. The lanes labeled 1, 2, 3 and 4 represent the time points of 114 h, 75 h, 38 h and 0 h, respectively. Right panel shows the quantification results. The first column was assigned a value of 1. (B) Examination of the rDNA copy number during replicative aging. Left panel, Southern blot with an rDNA probe, *ACT1* served as an internal control; right panel, quantification result, the rDNA copy number of wild-type (BWP17) young cells (column 4) is 55/cell as suggested (Jones *et al*., 2004). The lanes or columns labeled 1, 2, 3 and 4 represent the time points of 114 h, 75 h, 38 h and 0 h, respectively.

ERCs have been shown to accumulate during cellular aging in *S. cerevisiae*, so we wondered whether the extra-chromosomal rDNA molecules also accumulated along with the aging process in *C. albicans*. Thus, we performed PFGE and examined rDNA with Southern blot. In both wild-type strain and the two *sir2* deletion mutants, the extra-chromosomal rDNA molecules did not display any obvious changes as the replicative age increased (Fig. 5A). The extra-chromosomal rDNA molecules in hyphal cells seemed not to be as homogenous as that in yeast cells (compare the lane marked ‘SC5314 (yeast)’ with other lanes). We also examined the rDNA copy number in wild-type, *sir2Δ/SIR2* and *sir2Δ/sir2Δ* cells with different ages, and no increase was detected (Fig. 5B). All these findings further suggest that the extra-chromosomal rDNA molecules may not be associated with cellular aging of *C. albicans*.

### *Candida* Sir2 functions in the distribution of oxidatively damaged proteins during cell division

**Fig. 6:**
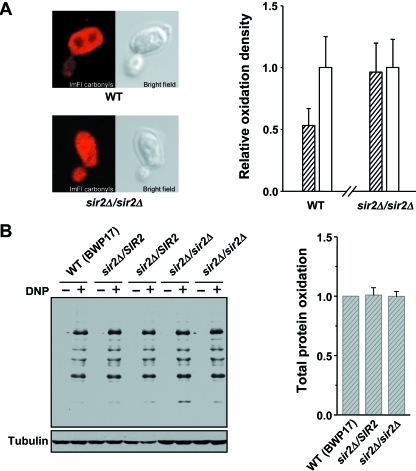
Analysis of oxidatively damaged proteins in wild-type and *sir2Δ* mutant cells. (A) Immunofluorescent detection of the distribution of oxidatively damaged proteins in mother and daughter cells. Left panel, typical budding cells visualized by Confocol microscopy. Right panel, oxidation density was quantified and compared between mother (open bars) and daughter (filled bars) cells. Fifty mother cells and corresponding daughter cells of each strain were used for quantification. Error bars are the standard deviations. Average oxidation density of daughter cells as compared with their mothers (assigned as 1): WT (0.53), *sir2Δ/sir2Δ* (0.96). (B) Western blot analysis of the overall protein oxidation in wild-type, *sir2Δ/SIR2* and *sir2Δ/sir2Δ* cells. Tubulin is used as an internal control. Right panel shows the quantification results. The level of total protein oxidation in wild-type strain was assigned as 1. Error bars represent standard deviations of two independent clones.

Since the correlations between Sir2 and extra-chromosomal rDNA molecules were not observed in *C. albicans*, and previous studies in *S. cerevisiae* elucidated that Sir2 regulates the asymmetric inheritance of oxidatively damaged proteins during cell division (Aguilaniu *et al*., 2003), we wanted to know whether *Candida* Sir2 also affects the distribution of oxidatively damaged proteins in mother and daughter cells. In wild-type strain, more oxidatively damaged proteins were retained in mother cells as compared to its daughter budding cell. In contrast, in *sir2Δ/sir2Δ* strain, the oxidatively damaged proteins were distributed evenly between mother and daughter cells (Fig. 6A). The result illuminates that cells fail to segregate oxidized proteins when Sir2 is absent. To ask whether the accumulation of oxidatively damaged proteins is increased in the *sir2Δ* mutants, we performed the carbonyl assay to detect the overall level of oxidatively damaged proteins. Interestingly, the overall level of oxidatively damaged proteins in *sir2Δ/SIR2*or *sir2Δ/sir2Δ*cells seemed not to be higher than that in wild-type cells (Fig. 6B). These data indicate that Sir2 controls the ability of mother cells to retain oxidatively damaged proteins during cell division. Our results are consistent with the previous findings in *S. cerevisiae* (Aguilaniu *et al*., 2003).

## Discussion

The unicellular eukaryotic organism, *S. cerevisiae* has been considered as one of the representative model organisms in aging research. Previous studies have indicated that *S. cerevisiae* and *C. albicans*, the two well-studied yeast, bear many resemblances genetically and biochemically (Berman & Sudbery, 2002). In our current work, we characterized *C. albicans*, the polymorphic fungus that has a finite lifespan (Fig. 1B). Like *S. cerevisiae*, the old *C. albicans* exhibits some aging-associated phenotypes, including morphologically larger in size (Fig. 2C), accumulation of glycogen and oxidatively damaged proteins (Fig. 3), and down-regulation of nascent protein synthesis (data not shown). In addition, the old *Candida* cells show no changes in telomere length and chromosome integrity (Figs S2 and S3). Moreover, *C. albicans* appears to share some conserved aging pathways with *S. cerevisiae*. For example, calorie restriction prolongs lifespan in *C. albicans* (Fig. 4A). Decreasing the dosage of Sir2 shortens lifespan, and overexpression of Sir2 leads to lifespan extension in *C. albicans* (Fig. 4B). These observations indicate that *C. albicans* could be considered as a new model system for aging study.

In *C. albicans*, deletion of one copy of *SIR2* affects the rDNA silencing (Fig. 4C), indicating that like *S. cerevisiae*, *Candida SIR2* is involved in the maintenance of silent chromatin. In *S. cerevisiae*, it has been shown that Sir2 may delay aging by repressing recombination in the rDNA loci so as to inhibit the formation of ERCs (Gottlieb & Esposito, 1989; Sinclair & Guarente, 1997). Interestingly, in *C. albicans*, the reduction of rDNA silencing in *sir2Δ*/*SIR2* mutant does not result in an increase of extra-chromosomal rDNA molecules (Fig. 4D). We speculate that the mechanism of regulating cellular aging by Sir2 in *C. albicans* may not be exactly the same as in *S. cerevisiae*. In fact, although the longevity-promoting role of Sirtuin family has been proved in a variety of species, including *S. cerevisiae*, *C. elegans*, and *Drosophila melanogaster* (Kaeberlein *et al*., 1999; Tissenbaum & Guarente, 2001; Rogina & Helfand, 2004), the extra-chromosomal rDNA molecule (circle) associated aging has not been observed in other organisms except *S. cerevisiae*. Therefore, it remains elusive whether the molecular mechanism by which the extra-chromosomal rDNA molecules (circles) cause aging is evolutionarily conserved. This idea is also supported by the observation that extra-chromosomal rDNA molecules do not accumulate in the aging *C. albicans* cells in either wild-type or *sir2* mutant strains (Fig. 5A). In addition, plasmids are indeed not stable in *Candida* cells (reviewed in De Backer *et al*., 2000). Thus, we postulate that the decrease of rDNA silencing in *sir2Δ/SIR2* cells accelerates aging through a mechanism other than promoting rDNA recombination to form extra-chromosomal rDNA molecules (circles) (Kennedy *et al*., 1997; Sinclair & Guarente, 1997).

Although it is not clear how Sir2 affect *C. albicans* longevity, oxidative damage accumulation seems to be a function of replicative age in *C. albicans*. It has been proved in *S. cerevisiae* that mother cells retain oxidatively damaged proteins during cell division, and the uneven distribution of these proteins between mother and daughter cells is not due to the different rate in production of reactive oxygen species or degradation of carbonylated proteins, but a result of asymmetric segregation of the oxidized proteins during cytokinesis in a Sir2-dependent manner (Aguilaniu *et al*., 2003). Given that in *C. albicans*, oxidatively damaged proteins accumulate with replicative aging (Fig. 3B), and evenly distribute in mother and daughter cells when Sir2 is absent (Fig. 6A), *Candida* Sir2 acts more likely as *Saccharomyces* Sir2 to protect the daughters from inheriting oxidative damage accumulated in the aged mothers. Therefore, Sir2 may promote longevity in *C. albicans* through regulating the distribution of oxidatively damaged proteins.

Besides the similar aging-associated phenotypes between *C. albicans* and *S. cerevisiae*, *C. albicans* is able to provide some advantages, potentiating it a complementary model. To prepare large amount of old cells seems to be a bottleneck for aging-related biochemical or genomics/proteomics study. Because *C. albicans* cells can grow as yeast form and hyphal form, which are dramatically different in shape and size, someone would be able to obtain a large amount of old cells. The method we have established for acquirement of old cells is relatively simple and effective. In addition, the separation procedure allows harvesting both young and old cells at discretionary time points, and provides an opportunity to perform the phenotypic time-course analysis of aging progression.

In conclusion, we have established a new cellular aging model of *C. albicans*, which allows large-scale isolation of replicative old cells. Our work also suggests that the extra-chromosomal rDNA molecules may not be associated with aging in *C. albicans.* This novel aging model will facilitate to find out novel aging markers and investigate the mechanisms of cellular aging.

## Experimental procedures

### Strains and growth conditions

The *C. albicans* strains used in this study are SC5314 (wild-type) and its derivative BWP17 (*ura3Δ::λimm434/ura3Δ::λimm434 his1::hisG/his1::hisG arg4::hisG/arg4::hisG*). To make *sgs1Δ/SGS1*or *sir2Δ/SIR2* strain, one copy of *SGS1*or *SIR2* was deleted, respectively, in BWP17 using a PCR-based gene disruption method (Wilson *et al*., 1999). The *sir2Δ/sir2Δ* strain was generated similarly on the basis of *sir2Δ/SIR2* strain. BES116 plasmid was used to construct the Sir2 overexpression strain (Feng *et al*., 1999). The plasmid contains two *ADE2* homologous arms (0.6 kb and 0.9 kb, respectively) for the recombination at *ADE2* locus and a *URA3* gene for nutrition selection. Integration of *SIR2* at *ADE2* locus was accomplished by transforming BWP17 with the linear fragment of BES116-SIR2 digested with *Asc*I. In addition to the entire coding region of *SIR2*, ∼1000 bp of upstream sequence and ∼500 bp of downstream sequence are included. A vector control was obtained by transforming the *Asc*I digested product of BES116 into BWP17. Yeast cells were propagated in YPD medium at 30 °C for routine culture. Hyphal cells were induced with an initial density of 10^6^ yeast cells mL^−1^ and cultured in YPD plus 15% bovine serum for 5 h at 37 °C.

### Replicative lifespan analysis

Replicative lifespan assay of yeast cells was performed as described previously (Kaeberlein *et al*., 2004). Unless otherwise specified, the replicative lifespan experiments were performed on yeast form cells. Lifespan of hyphal cells was determined as follows. Hyphal cells were separated from liquid culture (refer to the ‘Large-scale preparation of old and young cells’ section) and subjected to lifespan analysis immediately. A cohort of randomly selected hyphal cells was patched onto the lifespan plate and arrayed using a Singer MSM micromanipulator (Singer Instruments, Roadwater, Watchet, Somerset, UK). The budded yeast daughter cells from hyphal mothers were removed and counted every one to two generations. If a hyphal mother contains more than one cell, the average number of daughter cells was calculated. The plates were incubated at 30 °C during the day and stored at 4 °C at night. All lifespan experiments were carried out on standard YPD plates (2% glucose) except for CR analysis. Each experiment has been repeated at least twice, and data shown represent single experiment results. Statistical significance was determined by Wilcoxon rank-sum test, and significant differences were stated for *p* < 0.05.

### Calcofluor staining

The separated cells were stained with Fluorescent Brightener 28 (Calcofluor White M2R Tinopal UNPA-GX) (Sigma-Aldrich, St. Louis, MO, USA) to calculate the percentage of hyphal cells. One million cells were suspended in 1 mL of double-distilled H_2_O, sonicated to prevent clumping and resuspended in 95% ethanol for 1–2 h at 4 °C. After being washed with 150 µL of 1 m sorbitol, cells were resuspended in 150 µL of 1 mg mL^−1^ fluorescent brightener for 15 min at 4 °C. At last, the cells were washed three times with 1 mL of 1 m sorbitol and visualized under the fluorescent microscope (Zeiss Axioplan 2, Carl Zeiss Inc., Oberkochen, Germany).

### Separation of hyphal and yeast cells

Discontinuous sucrose density gradient was made by layering 30 mL of 30% sucrose at the bottom of a 50 mL centrifuge tube (Corning Inc., Corning, NY, USA) under 15 mL of distilled H_2_O. Five milliliter of a mixture of hyphal and yeast cells was loaded onto a sucrose gradient, and centrifuged at 150 *g* for 3–5 min. Hyphal cells stayed at the bottom of the tubes, and yeast cells stayed in the upper layer of the gradient. The upper layer supernatant was collected to pellet yeast cells by centrifugation (3000 *g*, 3 min). The bottom pellet was visualized under a Nikon Eclipse E200 (Tokyo, Japan) microscope to check the purity of separated hyphal cells. More sucrose gradient centrifugation(s) would be performed until no dissociative yeast cells were mixed in the hyphal population. Hyphal cells were finally pelleted by centrifugation (3000 *g*, 3 min).

### Large-scale preparation of old and young cells

A fresh colony was inoculated into YPD medium and incubated at 30 °C overnight. Hyphal cells were induced in the presence of 15% serum at 37 °C for 5 h, and then cultured in YPD for 2.5 h at 30 °C to produce yeast form cells. The newborn yeast cells in this short time were harvested by sucrose gradient centrifugation and used to induce the growth of young hyphae. The young hyphae were cultured in YPD at 30 °C to produce yeast form daughters continuously. During the cultivation sucrose gradient centrifugation was employed to get rid of yeast daughter cells, and hyphal mother cells were transferred to fresh YPD every 10–12 h. At the indicated time points, hyphal cells were separated thoroughly and subjected to replicative lifespan assay immediately.

### Iodine staining of glycogen

Hyphae of different ages were sonicated to break into single cells and then counted using hemacytometer. About 5 × 10^5^ cells for each sample were dotted on GF/C filters (Whatman International Ltd, Maidstone, UK) triply. The filters were stained for 2 min by exposing to iodine vapor at 37 °C, and photographed with a digital camera. Images were quantitated with Bio-Rad Quantity-One program (Bio-Rad Laboratories, Hercules, CA, USA).

### Determination of oxidatively damaged proteins

Total proteins were prepared using the glass beads protocol, and the protein concentrations were determined by Bradford (Bio-Rad Laboratories Inc., Hercules, CA, USA). Oxidative proteins were reacted with 2,4-dinitrophenylhydrazine in sodium dodecyl sulfate (Levine *et al*., 1994) and detected by Western blot with rabbit anti-2,4-dinitrophenyl antibody (Zymed Laboratories, South San Francisco, CA, USA).

### Separation of *C. albicans* chromosomes

The whole chromosomes were separated by PFGE as described previously (Chen & Fonzi, 1992).

### Determination of telomere length

*Candida albicans* telomere blot was performed as described (Singh *et al*., 2002). Genomic DNA was isolated using standard glass beads protocol and digested with *Nla*III and *Alu*I. Digested DNA was separated on a 0.9% agarose gel, transferred to Hybond-N+ membrane (GE Healthcare, Amersham Place, Little Chalfont, Buckinghamshire, UK), and hybridized with a 5′-end-labeled oligonucleotide (5′-GAAGTTAGACATCCGTACACCAAGAAGTTAGACATCCGTACACCAA-3′) containing two copies of the *C. albicans* telomere repeat.

### Detecting extra-chromosomal rDNA molecules and determination of rDNA repeat number

The extra-chromosomal rDNA molecules were detected by PFGE using a CHEF-DRIII system (Bio-Rad Laboratories). About 10^7^ cells were harvested and digested with 40 µL of 2 mg mL^−1^ Zymolyase 20 T (MP Biomedicals, LLC, Solon, OH, USA) at 37 °C, then mixed with 40 µL of 2% low-melting agarose (cool to 50 °C). Plugs were prepared at 4 °C by adding 80 µL of the samples into a mold chamber, then treated with LET buffer (0.5 m EDTA, 0.01 m Tris (pH 7.5), > 0.5%β-mercaptoethanol), NDS buffer (0.5 m EDTA, 0.01 m Tris (pH 7.5), 1% N-laurylsarcosine, 1 mg mL^−1^ Protease K) and 0.05 m EDTA for 16–24 h, respectively. The plugs were then filled in a 0.8% (w/v) agarose gel for electrophoresis. The running conditions for detecting extra-chromosomal rDNA molecules are as following: 0.5 × TBE buffer at 14 °C; constant voltage of 3.6 V cm^−1^; a 120S pulse interval for 20 h. DNA was visualized by ethidium bromide staining. After capillary transfer to Hybond-N+ membrane, the rDNA signals were detected by hybridization with a ^32^P labeled 1.7 kb PCR product of *C. albicans* 18S subunit of rDNA (forward primer: 5′-GGTTGATCCTGCCAGTAGTC-3′; reverse primer: 5′-GTTCACCTACGGAAACCTTG-3′). To determine the copy number of rDNA, genomic DNA was mini-prepared and digested with *Eco*RV and *Sal*I, and then separated on a 0.9% agarose gel. DNA was blotted to Hybond-N+ membrane and probed with rDNA. Subsequently the membrane was stripped and hybridized to a ^32^P labeled 1.1 kb PCR product of *C.* *albicans ACT1*(forward primer: 5′-ATGGACGGTGAAGTTGCTGC-3′; reverse primer: 5′-GTGGTGAACAATGGATGGACC-3′) for normalization. The copy number of rDNA was determined with Image Quant software. The young cells are assumed as 55 rDNA copies per cell (Jones *et al*., 2004).

### Immunofluorescent detection of oxidatively damaged proteins

The oxidatively damaged proteins were detected as described in (Aguilaniu *et al*., 2003). Cells were digested with Zymolyase in the digestion buffer (50 mm Tris-HCl, pH 7.5; 10 mm MgCl_2_; 1 m Sorbitol). Primary and secondary antibodies were Rabbit anti-DNP (Zymed Laboratories) and Cy™3-conjugated AffiniPure Donkey Anti-Rabbit IgG (H+L) (Jackson ImmunoResearch Laboratories, West Grove, PA, USA), respectively. The fluorescence density was quantified using Image-Pro Plus software.

## Supporting Information

**Fig. S1** Hyphal cells are separated from yeast cells by sucrose gradient centrifugation. The photo was taken under a Zeiss Axioplan 2 microscope.

**Fig. S2** Telomere length does not change during Candida aging. Two independent clones of each time point (0 h, 38 h, 75 h and 114 h) were examined. Lane Y, yeast form; lane H, hyphal form. TRFs represent terminal restriction fragments.

**Fig. S3** Electrophoretic karyotypes of *Candida* cells during aging. *C. albicans* chromosomes were separated by PFGE and stained with ethidium bromide.

**Fig. S4** Lifespan variations of different strains. (A) Lifespan analysis of three reference strains. Mean lifespan and sample size are: SC5314 20.3 (*n* = 50), BWP17 28.0 (*n* = 50), BWP17 (vector) 22.8 (*n* = 50). (B) Summary of mean lifespan of the wild-type strains SC5314 and BWP17. Each dot represents an independent lifespan experiment. The average of mean lifespan: SC5314, 20.9; BWP17, 26.9.
