# Supplementary figures and images for: Candida albicans, a distinctive fungal model for cellular aging study
Source: Aging Cell. 2008 Oct;7(5):746–57. doi: 10.1111/j.1474-9726.2008.00424.x (PMC2773528; doi:10.1111/j.1474-9726.2008.00424.x)

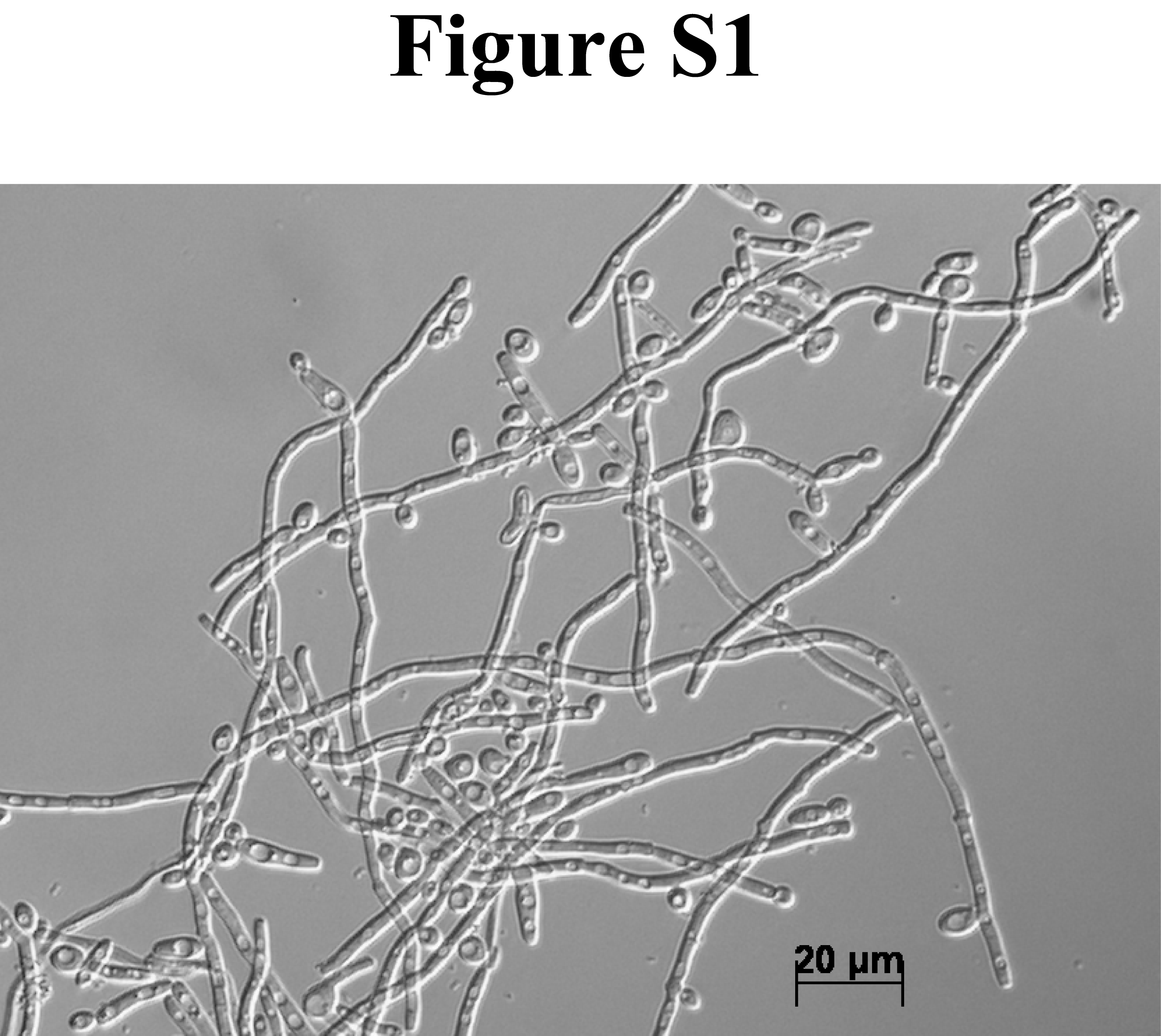

Supplement: Supplementary file 1 [file ace0007-0746-SD1.tif]

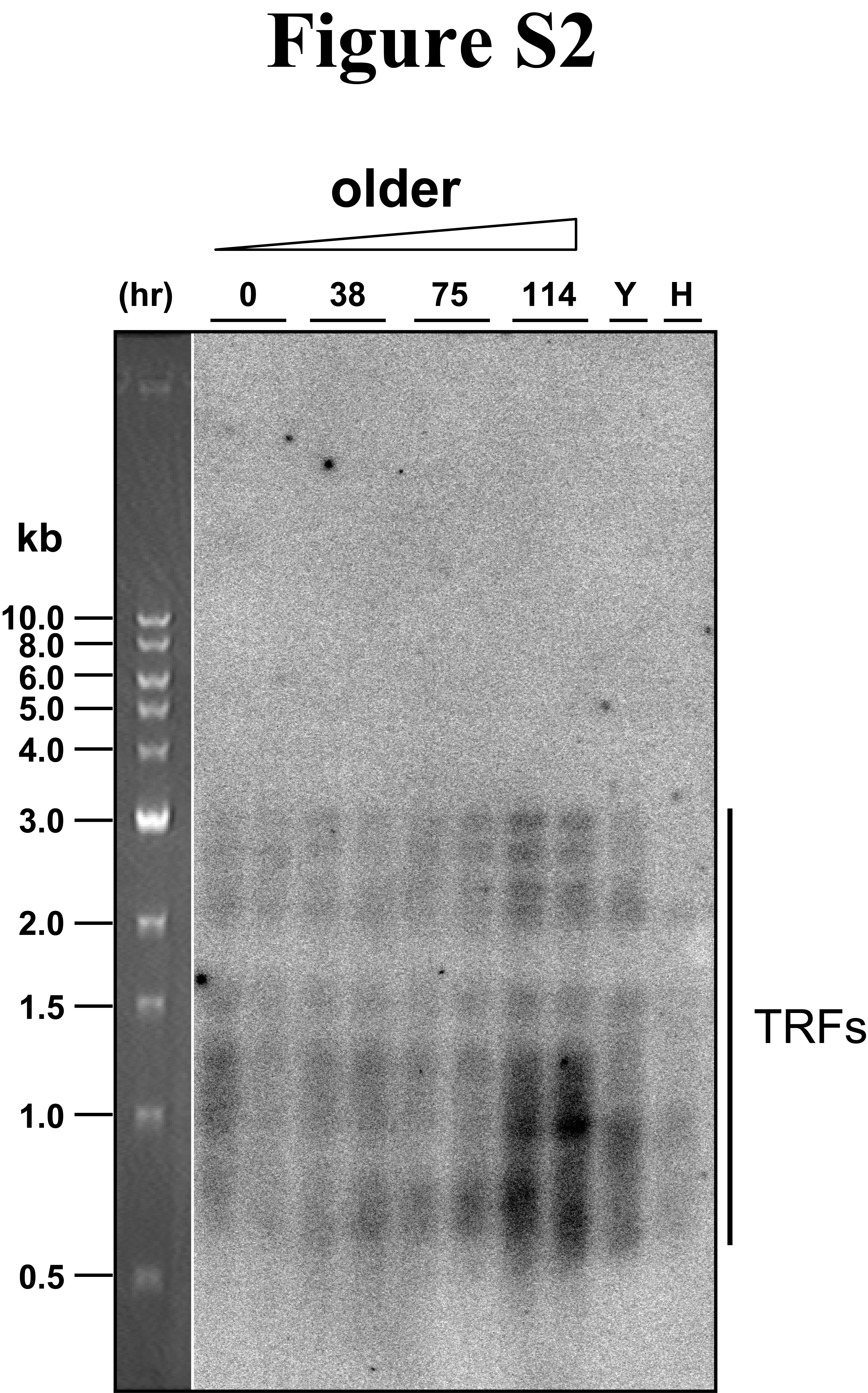

Supplement: Supplementary file 2 [file ace0007-0746-SD2.tif]

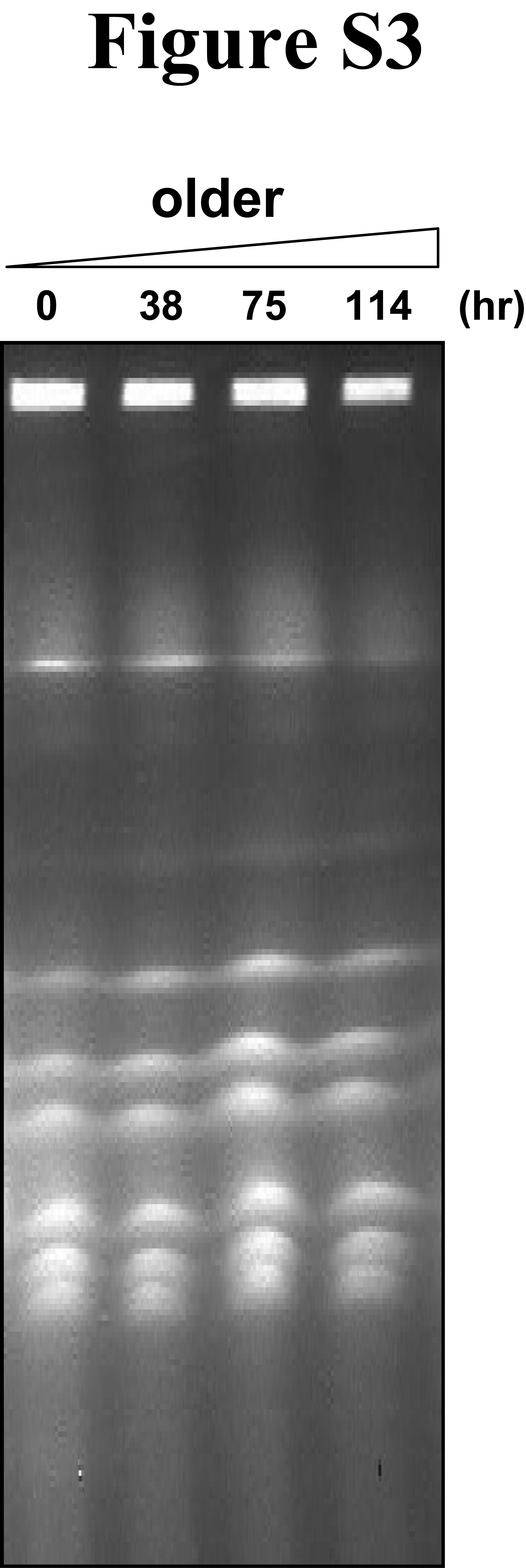

Supplement: Supplementary file 3 [file ace0007-0746-SD3.tif]

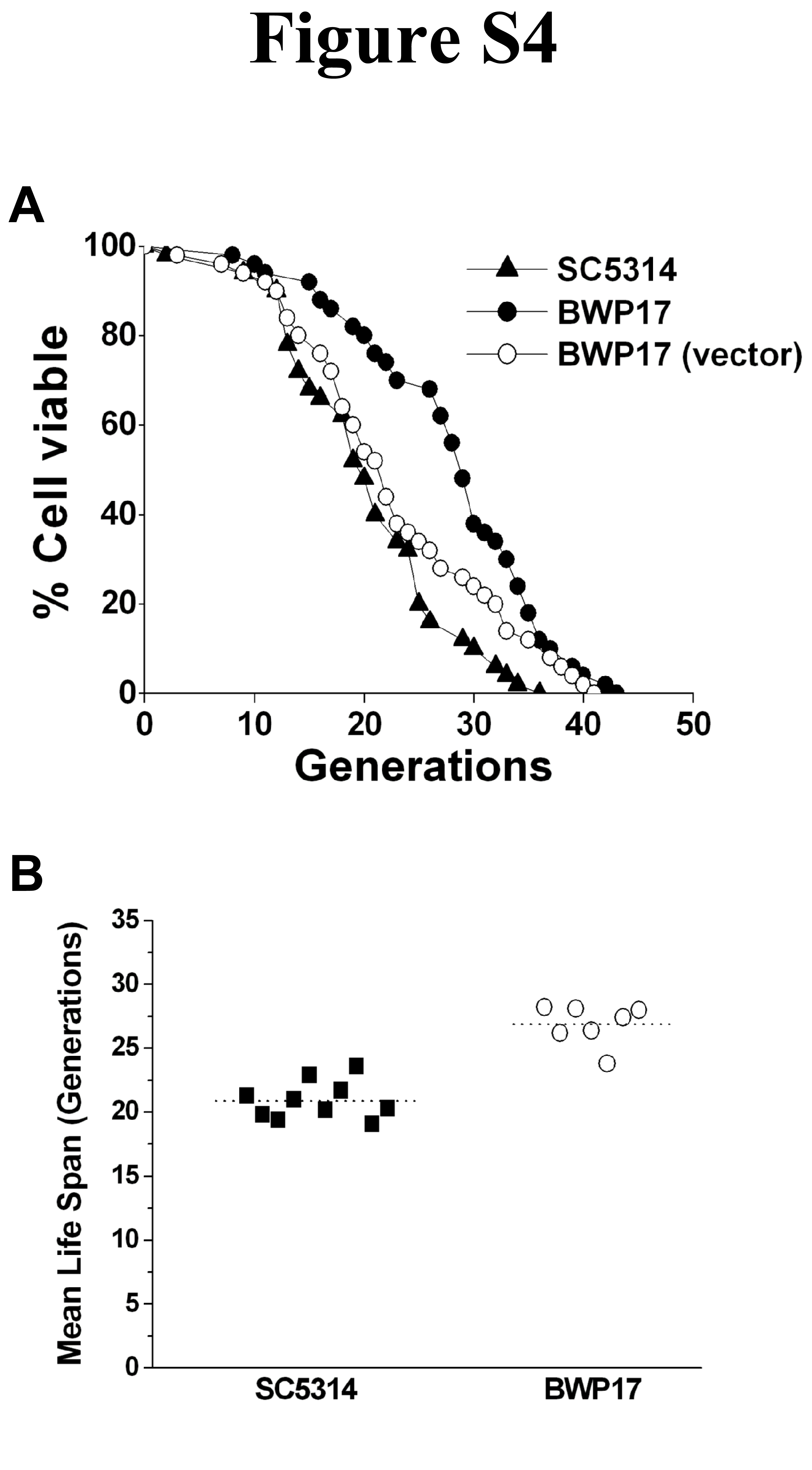

Supplement: Supplementary file 4 [file ace0007-0746-SD4.tif]
